# Identification of candidate transmission-blocking antigen genes in *Theileria annulata* and related vector-borne apicomplexan parasites

**DOI:** 10.1186/s12864-017-3788-1

**Published:** 2017-06-05

**Authors:** Laetitia Lempereur, Stephen D. Larcombe, Zeeshan Durrani, Tulin Karagenc, Huseyin Bilgin Bilgic, Serkan Bakirci, Selin Hacilarlioglu, Jane Kinnaird, Joanne Thompson, William Weir, Brian Shiels

**Affiliations:** 1 Institute of Biodiversity, Animal Health and Comparative Medicine, College of Medical, Veterinary & Life Sciences, University of Glasgow, 464 Bearsden Road, Glasgow, G61 1QH Scotland UK; 2 Present address: Laboratory of Parasitology and Parasitic Diseases, Department of Infectious and Parasitic Diseases, Faculty of Veterinary Medicine, University of Liège, Liège, Belgium; 3 Present address: School of Veterinary Science, University of Liverpool, Chester High Road, Neston, CH64 7TE, UK; 4 Faculty of Veterinary Medicine, Department of Parasitology, Adnan Menderes University, Batı Kampus, Işıklı, Aydın Turkey; 5 Institute of Immunology and Infection Research, School of Biological Sciences, Ashworth Laboratories, University of Edinburgh, The King’s Buildings, Edinburgh, EH9 3FL UK

**Keywords:** *Theileria annulata*, *Plasmodium*, *Babesia*, Bioinformatic screen, Transmission-blocking vaccine, 6-Cys domain

## Abstract

**Background:**

Vector-borne apicomplexan parasites are a major cause of mortality and morbidity to humans and livestock globally. The most important disease syndromes caused by these parasites are malaria, babesiosis and theileriosis. Strategies for control often target parasite stages in the mammalian host that cause disease, but this can result in reservoir infections that promote pathogen transmission and generate economic loss. Optimal control strategies should protect against clinical disease, block transmission and be applicable across related genera of parasites. We have used bioinformatics and transcriptomics to screen for transmission-blocking candidate antigens in the tick-borne apicomplexan parasite, *Theileria annulata*.

**Results:**

A number of candidate antigen genes were identified which encoded amino acid domains that are conserved across vector-borne Apicomplexa (*Babesia*, *Plasmodium* and *Theileria*), including the Pfs48/45 6-cys domain and a novel cysteine-rich domain. Expression profiling confirmed that selected candidate genes are expressed by life cycle stages within infected ticks. Additionally, putative B cell epitopes were identified in the *T. annulata* gene sequences encoding the 6-cys and cysteine rich domains, in a gene encoding a putative papain-family cysteine peptidase, with similarity to the *Plasmodium* SERA family, and the gene encoding the *T. annulata* major merozoite/piroplasm surface antigen, *Tams1*.

**Conclusions:**

Candidate genes were identified that encode proteins with similarity to known transmission blocking candidates in related parasites, while one is a novel candidate conserved across vector-borne apicomplexans and has a potential role in the sexual phase of the life cycle. The results indicate that a ‘One Health’ approach could be utilised to develop a transmission-blocking strategy effective against vector-borne apicomplexan parasites of animals and humans.

**Electronic supplementary material:**

The online version of this article (doi:10.1186/s12864-017-3788-1) contains supplementary material, which is available to authorized users.

## Background

Tropical theileriosis is a lymphoproliferative disease of cattle that occurs from Southern Europe and North Africa in the west, through the Middle East, Central Asia and Indian sub-continent, to China in the east. The disease is caused by infection of bovines with the tick-borne apicomplexan parasite *Theileria annulata* and is a severe constraint to livestock productivity. Tropical theileriosis can show acute and chronic forms; with acute disease characterised by fever, weakness and emaciation, swelling of superficial lymph nodes, destruction of the lymphoid system and pulmonary oedema. Death from acute theileriosis is common in susceptible *Bos taurus* cattle and can occur within 21–28 days. Overt theileriosis has been a major problem in endemic regions when European cattle have been imported to improve livestock productivity. However, it is likely that the economic loss from animals undergoing chronic disease or showing no apparent clinical signs (carriers) is greater than that due to overt disease. This was demonstrated in a Tunisian study where up to 38% of overall losses attributable to tropical theileriosis were associated with reduced milk production by carrier animals [1, 2]. Thus, to optimise economic output of cattle production in endemic regions, total control of theileriosis and related tick-borne disease (TBD) is required.

Current control measures include the use of acaricides, chemotherapy (primarily buparvaquone) and vaccination. Vaccination, with infected cell lines that develop attenuated virulence upon long-term culture, has been utilised in several countries [2, 3]. These vaccines can provide protection against clinical disease in the field but do not prevent establishment of carrier status. Thus, vaccination does not negate economic loss or the possibility of onward transmission from immunised carrier animals. In addition, for live vaccines there are potential risks of contamination with viral pathogens and reversion to virulence, and good quality control and a cold chain are required for effective delivery. Due to these disadvantages, plus recent reports of resistance to buparvaquone [4] and problems with continued use of acaricides (reviewed in [5]) there is a clear need for research into alternative, complementary control strategies.

An obvious strategy to control tropical theileriosis, and other TBD, is to prevent onward transmission of the pathogen by the tick vector. The efficacy of targeting ticks to block disease transmission is well known and has been validated by modelling studies, risk factor analysis and deployment of acaricides [6–8]. Use of acaricides, however, has an environmental impact and leads to selection of acaricide-resistant ticks [9]. The potential for anti-tick subunit vaccines to control tick infestation and decrease acaricide use has been demonstrated [10], with studies on the hidden gut antigen of *Boophilus microplus* (BM86) providing a paradigm model. Vaccination of cattle using the BM86 orthologue of *Hyalomma anatolicum anatolicum* (HAA86) showed that the tick gut antigen partially protected against homologous tick challenge and also reduced transmission of *Theileria annulata* [11]. In addition to targeting the tick, the potential of targeting surface antigens of the *Theileria* sporozoite and piroplasm stages to block transmission has been investigated. Antibodies against SPAG1 can effectively block invasion of the leukocyte by the sporozoite, while a response against the immunodominant Tams1 antigen has been implicated in blocking transmission of predominant genotypes [12, 13]. However, both these antigens show a degree of antigenic diversity in the parasite population that restricts their effectiveness as vaccine candidates [14–17]. This is particularly pertinent for Tams1 with identification of many allelic sequences, evidence of domain shuffling to generate molecular mosaics and the breakthrough of under-represented genotypes encoding variant Tams1 alleles when a carrier infection is transmitted through ticks [13, 14].

In order to circumvent antigenic diversity, proteins that perform a function that requires polypeptide domains to be invariant in the parasite population could be targeted. A potential advantage of selecting conserved protein domains is that they may be effective across a range of vector-borne diseases, by targeting processes or antigens common across related pathogens. One process of vector-borne Apicomplexans (*Babesia*, *Plasmodium* and *Theileria*) that could involve molecules conserved across genera is the sexual phase of the life cycle, which is obligatory for transmission of these parasites through their arthropod hosts. Proteins that function in the sexual phase and have potential to induce a transmission-blocking response against *Plasmodium spp*. have been identified. Surface antigens such as Pfs230, Pfs48/45, and Pfs25 are known to induce an immune response in vaccinated mammalian hosts that blocks transmission through the mosquito, thus demonstrating the feasibility of single or multi-subunit transmission blocking vaccines (TBVs) [18–22]. A considerable number of potential TBV candidates that perform functions required during the mosquito phase of the *Plasmodium* life-cycle have since been characterised (reviewed in [23]).

In the present study, a screen for parasite genes encoding antigens with the potential to induce a transmission-blocking response against *T. annulata* was conducted. A combination of bioinformatic prediction and transcriptional expression profiling was used to obtain a panel of candidates, a number of which have homologues across genera of related vector-borne Apicomplexa. Analysis of the expression levels of four candidate genes in the tick vector, together with investigation of predicted antigen diversity (*in silico*) provides evidence that development of transmission-blocking strategies which can operate across related vector-borne Apicomplexa may be possible.

## Methods

### Bioinformatic screening

A bioinformatic approach was used to identify *Theileria annulata* genes encoding proteins predicted to be located on the parasite surface using information representing 3772 genes contained in the genomic databases, GeneDB (http://www.genedb.org/Homepage/Tannulata) and EuPathDB (http://eupathdb.org). Genomic annotation data was downloaded using the ‘List Download’ feature of GeneDB. Candidate genes encoding putative surface antigens were selected on the basis of motifs predicted to be present on the encoded protein, namely a signal peptide, a GPI-anchor signal and/or one or more transmembrane domains. Database prediction for signal peptide (SignalP 2.0 HMM), GPI-anchor signal (DGPI v2.04) and transmembrane domains (TMHMM Server v2.0) were utilised using default settings. For *TA20855* and related homologues, sequences were also analysed using the SignalP 3.0 Server (http://www.cbs.dtu.dk/services/SignalP-3.0/) and SignalP 4.1 Server (http://www.cbs.dtu.dk/services/SignalP-4.1/). Integral membrane proteins with multiple predicted transmembrane domains were excluded. A subset of *Theileria annulata* genes that display elevated levels of mRNA expression from the macroschizont to the piroplasm stage of the life cycle in the vertebrate host were identified using a published microarray dataset [24]. Hierarchical clustering of log2-transformed gene expression levels and profiles of gene expression values across stages (sporozoite to piroplasm) were performed using DNASTAR Array Star3 software, as described [24]. The NCBI database was BLAST searched (https://blast.ncbi.nlm.nih.gov/) to identify homologues of candidate genes in other vector-borne Apicomplexan parasites, namely *Plasmodium spp*., *Theileria spp*. and *Babesia spp*.

### Revised annotation of *TA20855* and *TA19820*

Following alignment of homologs across genera for *T. annulata* genes *TA20855 and TA19820* it was observed that conservation of amino acid sequence observed for other apicomplexa was not obtained with sequence predicted for the *T. annulata* genes. Analysis of the gene DB entry sequence, however, showed both genes contained multiple introns and sequence with greater identity to the predicted amino acid sequence conserved across genera. An altered open reading frame was then identified and used to generate a revised amino acid sequence with greater conservation across genera. In order to verify that the revised predicted amino acid sequences were accurate, we used available next generation sequencing data. RNA-seq reads generated from sheep B-cells inoculated with *T annulata* stabilate (Ta Ankara, stabilate 89) for another experiment were kindly provided by Prof Ivan Morrison (Roslin Institute, University of Edinburgh). These RNA reads were of sufficient depth to provide coverage across the predicted *TA20855* and *TA19820* genes. Using the Bowtie 2 sequence aligner [25] RNA-seq reads were aligned to the predicted CDS of *TA20855* and *TA19820* provided on GeneDB. As expected, analysis of the created contigs revealed significant gaps in coverage, suggesting incorrect annotation and the presence or absence of introns.

The revised predicted amino acid sequences (designed to maximise orthology across Apicomplexa) were then aligned with the gDNA sequences (using Genewise Protein-nucleotide alignment software) to generate a new gene model and predicted CDS for both genes. The RNA seq reads were then mapped to the new predicted CDS sequences using Bowtie 2. The revised contigs showed much greater overlap between reads and coverage, including regions where incorrectly annotated introns and exons were responsible for the frame shifts in the original GeneDB gene models. Further revision to the predicted CDS was made to close gaps in RNA-seq coverage caused by other unidentified intron or exons, resulting in complete coverage and overlapping mRNA reads across the CDS (see Figs. A and D in Additional file 1). For *TA20855*, the final gene model results in 11 exons in contrast to 8 in the GeneDB model, while for *TA19820* the revised gene model results in the lengthening of 3 introns, and the inclusion of one more intron in contrast to the GeneDB entry (see Figs. B and E in Additional file 1). The revised mRNA sequence for both genes is extremely similar to the reference genome sequence, with only a handful of SNPs.

### qRT-PCR on selected candidate genes for a time course of *T. annulata* infected ticks

Four thousand one hundred ticks (*Hyalomma anatolicum anatolicum*) were fed on a calf infected with *T. annulata* Ankara sporozoite stabilate A10/BT (applied to the calves on Day 8 to Day 12 post-infection) with the parasitaemia peaking at 4% on Day 14. Engorged ticks were collected (stored at 15 °C, until collection of all ticks post-detachment) and then incubated at 28 °C for 2, 6, 10 and 15 days, followed by freezing in RNAlater® (Thermo Fisher Scientific) at−80 °C. These time-points represent early events in gametocyte maturation (Day 2) together with gamete (Day 6–10), zygote (Day 10) and kinete (Day 15) production, as reported previously [26, 27]. 400 frozen ticks for each time-point were crushed in liquid nitrogen and RNA extracted using TRIzol Reagent (Invitrogen) following the manufacturer’s protocol. Four candidate genes (*TA10955*, *TA17050*, *TA03640* and *TA20855*) were selected for qRT-PCR based on bioinformatically predicted characteristics, microarray gene expression profile and detection of orthologues in other vector-borne Apicomplexa. Primers were designed (Additional file 2) and qRT-PCR was performed as described previously [24]. Briefly, 500 ng of total RNA from each sample was used to synthesise cDNA using the Affinity Script cDNA Synthesis Kit (Agilent Technologies) and Oligo-dT as primer.

One μl cDNA for each sample was used for qRT-PCR, using the Brilliant III Ultra-fast SYBR®Green qPCR Master mix (Agilent technologies) and the Stratagene Mx3005P system. Comparative quantitative analysis of gene expression across time-points was performed using Stratagene MxPro Software, with RNA from a merozoite Day 8 culture used as the calibrator. HSP70 (*TA11610*) and HSP90 genes (*TA10720*) were utilised as controls for constitutive expression, based on their transcriptional profile through the life-cycle [24]. Differences in mean fold-change between time-points in candidate gene expression level were tested using Student’s *t*-test; *P*-values obtained are denoted in the Results section and in Figure Legends.

### Allelic dN/dS and epitope mapping for selected candidate genes

Analysis of allelic sequences generated from DNA samples from different *T. annulata* isolates from four different geographic origins was performed, with ratios of dN/dS computed to screen for evidence of diversifying positive selection for amino acid substitution on a codon-by-codon basis. The DNA samples were: *T. annulata* Ankara (Turkey), Hissar (India), 9A (Tunisia) and UmBanein24 (Sudan). PCR for genes of interest was performed on DNA from each of the four isolates and the resulting PCR amplicons were cloned and sequenced. Primers were specifically designed to amplify almost the entire length of *Tams1* (*TA17050*), putative papain-family cysteine protease (*TA10955*), and hypothetical protein *TA20855* (Additional file 2). *TA03640* was too large for the whole gene to be sequenced (>3000 bp), so for preliminary analysis two shorter fragments (~1500 bp and 2000 bp) were amplified, and five test sequences for each fragment generated. As this showed the second segment of the gene to be more polymorphic than the first, this region was chosen for further investigation of allelic polymorphism. Optimum annealing temperatures for each primer pair were determined (Additional file 2) and, to minimise the chance of PCR error in amplicons, *Pfu* Turbo DNA polymerase (Agilent Technologies) or KAPA HiFi (Kapa Biosystems,) polymerase was used in the PCR reaction, according to the manufacturer’s guidelines. PCR products were cloned into pCR®4Blunt-Topo vector (Invitrogen) and used to transform competent *E. coli*, using standard methodology. For *TA17050* and *TA10955*, twelve colonies from each isolate were selected and inserts sequenced in both directions (96 total sequences for each gene) by Genoscreen (Lille, France). For *TA20855* and *TA03640*, six colonies from each isolate were selected and sequenced in both directions (48 sequences for each gene, in total) by Eurofins (Berlin, Germany). The assembled sequences were translated and aligned to the GeneDB reference amino acid sequences (GenBank accession n° XP_953719, XP_953243, XP_954368) using CLC Genomics Workbench software and polymorphic sites identified. The datasets of allelic sequences were then used to estimate the ratio of non-synonymous to synonymous base-pair substitutions (dN/dS) for each codon in each gene and for the entirety of the selected gene or region using the SLAC algorithm of the online Datamonkey program (http://www.datamonkey.org [28]). The SLAC method is a conservative method for calculation of dN/dS that prevents overestimation of positive selection [29]. Finally, we used the Bepipred linear B-cell epitope prediction tool (http://tools.immuneepitope.org/bcell/) [30] to predict areas of each gene that could form B-cell epitopes. Data from both types of analysis were then overlaid to visualise any regions for each candidate gene where evidence for selection of amino acid substitution and prediction of a B cell epitope overlapped.

## Results

### Bioinformatic and transcriptomic profile analysis identifies *T. annulata* transmission-blocking candidate genes

To screen for *T. annulata* candidate genes encoding proteins that may be expressed by life cycle stages present in the tick vector a combined genomic and transcriptomic approach was taken. A screen of available genomic data was used to identify genes encoding proteins with a predicted signal peptide domain together with a GPI anchor domain, resulting in a list of 44 genes. Seven genes did not have direct orthologues in the closely related *Theileria parva* and were removed from the list. A further seven genes encoding proteins with multiple transmembrane domains were also removed, as these were considered likely to be integral membrane proteins, and so potentially less suitable as transmission blocking candidate antigens, leaving 30 candidate surface protein encoding genes. Microarray-derived transcriptomic data across all bovine life-cycle stages together with the tick-derived sporozoite stage was then analysed for these candidate genes [24]. From this, a subset of genes was selected which displayed an expression pattern that indicated rising mRNA levels from macroschizont through to piroplasm, the stage that is taken-up by the tick vector. Genes were selected on the basis of an absolute fold-change of greater than 2 between the macroschizont and merozoite and/or piroplasm stages. This resulted in a subset of 12 candidate genes (Table 1).

**Table 1:** Bioinformatic characteristics of 12 transmission blocking candidate (surface) antigen genes

*T. annulata* ID	Chr	Product	Annotation	Signal peptide	GPI anchor	TMD	Macro to mero FC	Macro to piro FC	Mero to piro FC	T. parva ID	dN/dS	Protein identity	Nucleotide identity
*TA02580*	3	Hypothetical protein	–	✓	✓	0	2.14	2.74	1.28	TP03_0040	0.0732	28.07	46.00
*TA02585*	3	Hypothetical protein	–	✓	✓	0	1.32	2.34	1.77	TP03_0039	0.0127	31.29	47.24
*TA03640* ^*^	3	Hypothetical protein	Sexual stage antigen (Pfam:PF07422)	✓	✓	0	3.55	4.14	1.17	TP03_0268	0.2529	64.17	73.18
*TA03755*	3	Sporozoite surface antigen (SPAG)	P67 sporozoite (Pfam:PF05642)	✓	✓	1	4.23	4.29	1.01	TP03_0287	0.3260	49.85	63.60
*TA10955* ^*^	4	Putative papain-family cysteine protease	Cysteine-type peptidase activity (GO:0008234)	✓	✓	0	3.42	4.61	1.35	TP04_0598	0.0904	85.50	83.48
*TA13810*	2	Putative ts-chitose type 23 kDa piroplasm surface-like protein	Orthologous to *T. sergenti* merozoite surface antigen	✓	✓	1	2.55	1.97	−1.29	TP02_0551	0.1638	83.41	84.57
*TA13825*	2	Hypothetical protein	–	✓	✓	1	12.40	11.00	−1.13	TP02_0553	0.4276	49.76	64.30
*TA16005*	2	Hypothetical protein	Domain of unknown function DUF529 (Pfam:PF04385)	✓	✓	1	1.87	3.09	1.65	TP02_0950	0.3317	62.59	75.61
*TA16565*	1	Hypothetical protein	–	✓	✓	0	1.60	2.27	1.41	TP01_1144	0.2704	74.68	79.69
*TA17050* ^*^	1	Merozoite-piroplasm surface antigen Tams1	Merozoite antigen (Pfam:PF02488)	✓	✓	1	2.62	2.64	1.01	TP01_1056	0.2751	72.86	77.38
*TA17220*	4	Hypothetical protein	Domain of unknown function DUF529 (Pfam:PF04385)	✓	✓	1	1.66	2.81	1.69	TP04_0030	0.3037	51.59	68.65
*TA20855* ^*^	1	Hypothetical protein	Similarity to *P. yoelii* (SWALL:EAA20932) and *P. falciparum* (SWALL:Q8IE86)	✓	✓	0	2.57	2.63	1.03	TP01_0412	0.085	80.43	77.72

Bioinformatic prediction of surface location of 12 candidates genes with significant fold change in gene expression levels between macroschizont and merozoite/piroplasm stages. Candidates selected for allelic sequencing are marked with an asterisk and protein and nucleotide identity are to the putative *T. parva* orthologue

**Fig. 1:**
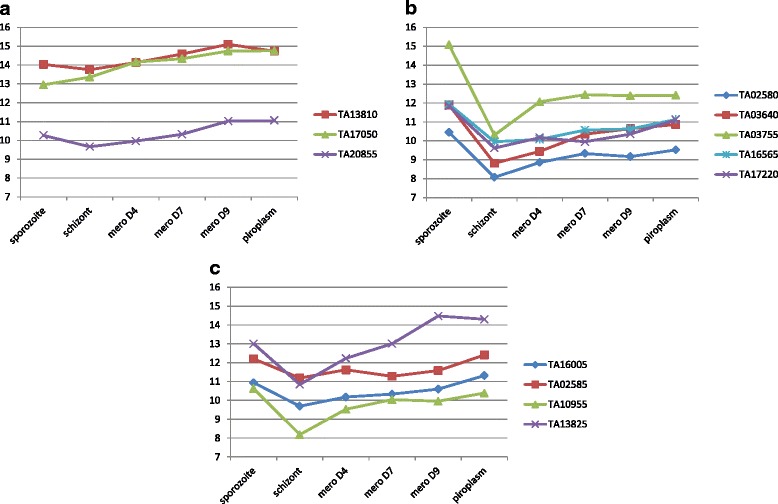
Gene expression profile of 12 transmission-blocking candidates. Microarray expression profiles (**a**), (**b**) and (**c**) of *T. annulata* candidate genes in sporozoite, schizont, merozoite Day 4, Day 7, Day 9 and piroplasm. Expression is depicted on a log_2_ scale

Expression profiles were constructed across all stages, for which data was available, with candidate genes grouped into three profile types (see Fig. 1). In the first profile (**a**): genes *TA13810*, *TA17050* and *TA20855* showed elevation of expression from macroschizont through to the merozoite/piroplasm and the level of expression in the sporozoite was similar to that of the macroschizont (<2 fold difference). In the second profile (**b**): genes *TA02580*, *TA03640*, *TA03755*, *TA16565*, *TA17220*, showed elevation of expression through to the merozoite/piroplasm stages and had a significantly higher level of expression (>2 fold) in the sporozoite relative to the macroschizont stage, and the expression level in the sporozoite was higher than that for the merozoite/piroplasm. In the third profile (**c**): genes *TA02585*, *TA16005*, *TA10955* and *TA13825* showed expression levels that were higher in sporozoite than in macroschizont and either comparable between sporozoite and piroplasm or lower in the sporozoite. Within these profiles two genes previously considered as transmission-blocking candidates were identified. *Tams1* (*TA17050*) showed expression consistent with profile (**a**), while *SPAG1* (*TA03755*) showed expression consistent with profile (**b**). It was concluded that these profiles indicate the potential for the gene to be expressed, either transiently in the tick following a blood meal, or at an elevated level that is coincident with the development of stages within the tick vector.

### Identification of candidate genes showing domain conservation across vector-borne Apicomplexa

To determine if any of the candidate genes are predicted to possess domains that perform a conserved function, their entries in GeneDB were examined and BLAST analysis for homologues in other Apicomplexa was carried out. *Tams1* (TA17050) and *SPAG1* (*TA03755*) have been characterised extensively with orthologues identified across the *Theileria* genus, they have no known domains that show conservation across the vector-borne Apicomplexa.

*TA02580* and *TA02585* encode putative surface proteins of unknown function with respective orthologues only identified in *T. parva. TA13810* was identified as the direct orthologue of the gene encoding the ts-chitose type 23 kDa piroplasm surface protein of *T. sergenti* [31] and is conserved across bovine *Theileria spp*.; an orthologue was not identified for other vector-borne Apicomplexa. Similarly, orthologues of *TA13825* were only identified in *Theileria ssp.* (*T. parva*, *T. orientalis/buffeli* and *T. equi*) and showed similarity to the 23 kDa piroplasm surface protein. *TA16005* encodes a protein of unknown function that is also restricted to *Theileria* species. *TA17220* has a probable orthologue in *T. parva* and shows similarity to an uncharacterised predicted protein in *T. orientalis. TA16565* is annotated as an uncharacterised surface protein with orthologues in both *Theileria* and *Babesia* genera (E-value-1.5E-17, *B. bovis*; - 9.5E-19, *B. bigemina*; 3.2E-25, *B. microti*).

**Fig. 2:**
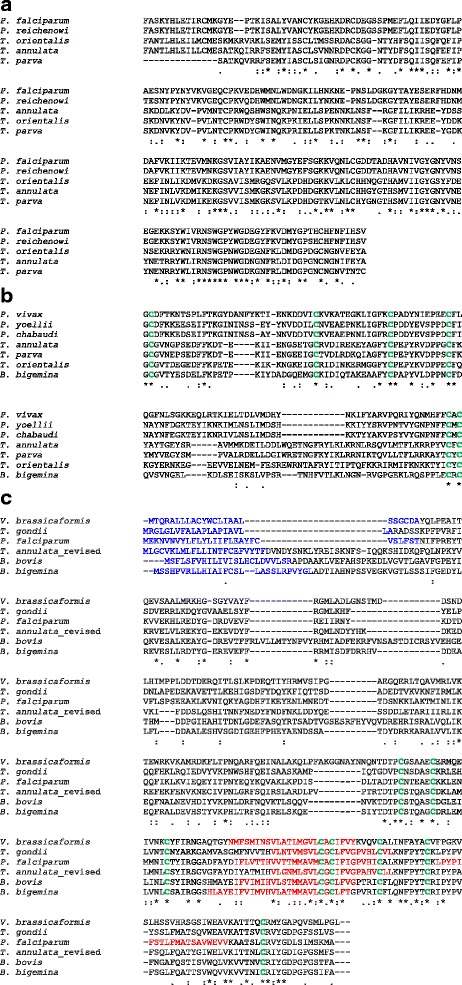
Protein alignments with related Apicomplexan genera. (**a**) Alignment of the conserved papain family cysteine protease domain of Serine Repeat Antigen (SERA)-like Proteins from : *P. falciparum* (PF3D7_0207600), *P. reichenowi* (PRCDC_0206900), *T. orientalis* (TOT_040000333), *T. annulata* (*TA10995*), *T. parva* (TP04_0598). (**b**) Alignment of the highly conserved s48-45 superfamily 6-cysteine domain from sequences of *TA03640* homologues with conserved cysteine residues in *green*: *P. vivax* (PVP01_113600), *P. yoellii* (PYO3100), *P. chabaudi* (PCHAS_0111600), *T. annulata* (*TA03640*), *T. parva* (TP03_0268), *T. orientalis* (TOT_030000578), *B. bigemina* (BBBOND_0402900). (**c**) Alignment of the highly conserved 8-cysteine domain region of (revised) *TA20855* homologues with predicted signal peptides (*blue*), transmembrane helices (*red*) and cysteine residues (*green*) highlighted: *V. brassicaformis* (VBRA_17621), *T. gondii* (*TGME49_321580*), *P. falciparum* (*PF3D7_1322900*) *T. annulata*_revised (*TA20855*), *B. bovis* (*BBOV_IV006060*), *B. bigemina* (*BBBOND_0208520*)

*TA10955* is annotated in GeneDB as encoding a putative papain-family (clan CA) cysteine protease (Pfam: PF00112, E-value = 1.86E-12) with a signal peptide and GPI anchor. BLAST analysis identified similarity (30% identity, 49% similarity) to the Serine repeat antigen 5 of *Plasmodium falciparum* that covers the predicted peptidase domain (218–476) of TA10955. Conservation of the domain (see Fig. 2a) was found in predicted proteins of related *Theileria* species (*T. orientalis* and *T. parva*) as previously reported [32], but not in *Babesia* or *T. equi*.

Gene *TA03640* is annotated as encoding a hypothetical protein with a signal peptide and GPI anchor. It is also annotated as encoding an s48_45 domain between aa 1020-1135 (Pfam: 07422, E-value-1.2E-17) found in the 6-cys family of *Plasmodium* surface proteins (e.g. Pfs 48/45 and Pfs 230) that play an important role in gamete fertilisation in *Plasmodium* [33, 34]. The domain contains 6 conserved cysteines that form 3 disulphide bridges necessary for correct protein folding. The s48/45 domain is conserved across the vector-borne Apicomplexa with orthologues present in *Theileria* and *Babesia* species, as well as *Plasmodium*. The alignment represented in Fig. 2b shows strong positional conservation of the 6 cysteines of the *Theileria* domain with orthologues in *Plasmodium* and *Babesia*.

*TA20855* is annotated in Gene DB as encoding a hypothetical surface protein of 297 aa with similarity to *Plasmodium* hypothetical proteins. Clear homologues with significant similarity (>50%) were identified by BLAST across the Apicomplexa (*Babesia*, *Plasmodium*, *Toxoplasma*, *Hammondia*), with the highest level of similarity spanning a region containing conserved cysteine residues. However, based on identity of predicted amino acid sequences across other genera, compared to that identified for the *Theileria* orthologues, it was concluded that the original annotation of intron exon junctions in *TA20855* on GeneDB predicted an incorrect open reading frame, with the *TA20855* sequence diverging from those of related genera at aa 255. A revised gene model (based on homology of predicted aa sequence across genera) encoding a protein of 289 aa was then validated using available RNA seq data, with complete coverage of the revised polypeptide coding sequence obtained (see Additional file 1). Using the revised gene model, homology over a region spanning aa 128–282 (58% similarity; minimum E-value 2E-18) was found across genera of Apicomplexa, and *Vitrella brassicaformis*, a chromerid that evolved from a common ancestor shared with the Apicomplexa [35]. This region of homology contains 8 positional conserved cysteine residues and spans at least one predicted transmembrane (TM) helix, with a second more C-terminal helix predicted in some instances (depending on the sequence modelled or the algorithm used; see Fig. 2c and Additional file 1). Both these predictions, plus the prediction of a cleaved signal peptide (*T. annulata*; *B. bovis*, *B. bigemina*, *Vitrella brassica* (using both SignalP 3.0 and 4.1) and *T. gondii* (SignalP 3.0 but not 4.1)), indicate that the majority of the encoded polypeptide (a helical rich region) is to the extracellular side of the membrane. For *Plasmodium* polypeptides, while a cleaved signal peptide is predicted by SignalP 3.0, an alternative model with the helical rich region on the cytoplasmic face is also indicated, as a transmembrane helix is also denoted within the putative signal peptide region in their EuPathDB entries. Thus, gene *TA20855* is likely to encode a membrane protein that is conserved across related vector borne genera and was present in a common ancestor of the apicomplexans and chromerids.

### Elevated expression of candidate genes in tick stages of *T. annulata*

To assess potential expression of selected candidate genes in tick stages of *T. annulata*, qRT-PCR was performed on RNA representing a developmental time-course after engorgement of ticks on a piroplasm-infected animal. Four genes were selected representing the (**a**) (*TA17050* and *TA20855*), (**b**) (*TA03640*) and (**c**) (*TA10955*) microarray expression profiles. These genes include a *Theileria*-specific candidate (*TA17050*), a candidate that showed homology with *Plasmodium* proteins (*TA10955*) and two candidates (*TA03640* and *TA20855*) with homologues present in vector-borne Apicomplexa.

**Fig. 3:**
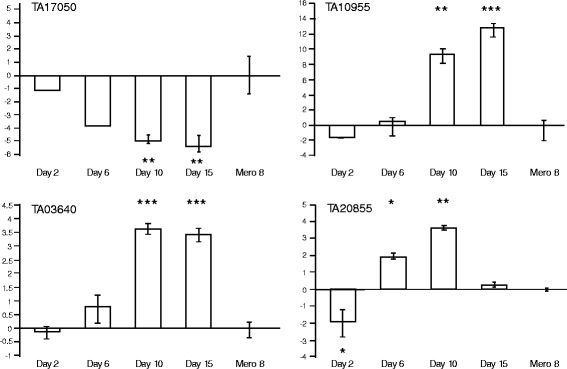
*qRT-PCR of candidate genes in tick stages.* Quantitative RT-PCR expression analysis of RNA from *T. annulata* infected ticks generated at Day 2, Day 6, Day 10 and Day 15 post-detachment, relative to *T. annulata* merozoite Day 8 (calibrator) for: *Tams1* (*TA17050*); putative papain-family cysteine protease (*TA10955*); Pfs 48/45 6-cys domain encoding gene *TA03640* and 6-Cys like gene *TA20855.* * above (positive) or below (negative) error bars denote degrees of significant difference (* *p* < 0.05, ** *p* < 0.01, *** *p* < 0.0001) between fold-change at a time-point relative to merozoite calibrator RNA. Expression is depicted on a log_2_ scale

As illustrated in Fig. 3, the expression profile for *TA17050* (*Tams1*) showed a drop in expression at day 2, relative to the merozoite stage calibrator RNA (2.1-fold, absolute), which continued as the infected tick time-course progressed to Day 15 (39-fold reduction, absolute). *TA10955* (the putative papain-family cysteine protease gene) showed a decrease (>3-fold, absolute) in expression at Day 2 post-detachment of ticks relative to merozoite RNA (see Fig. 3). However, in contrast to *Tams1,* from day 2 onwards, expression of *TA10955* increased as the tick time-course progressed, with a marked significant (*p* < 0.001) elevation at Day 15 (>7000 fold absolute at Day 15, relative to merozoite RNA).

For *TA03640*, significant elevated expression was not detected at the early time-points. By Day 10, however, expression levels were increased significantly relative to merozoite and Day 2 (>13 fold, absolute, *p* < 0.0001), and this was sustained at Day 15 (Fig. 3). A related expression profile was obtained for *TA20855* with expression significantly elevated at Day 6, relative to Day 2 (*p* = 0.002), and a further increase (>3-fold, absolute, *p* < 0.001) at Day 10 relative to Day 6 (Fig. 3). However, unlike *TA03640*, a significant fall (*p* < 0.001) in expression between Day 10 and Day 15 occurred (>10-fold, absolute, decrease) to a level below that of the Day 6 time-point (>3-fold, absolute, decrease). To compare expression profiles for the *Plasmodium and Toxoplasma* homologues of *TA20855*, data available in EuPathDB was mined. This demonstrated that for all *Plasmodium* homologues for which data is available, RNA is up-regulated in late stage (V) gametocytes, indicating a putative role in transmission via the mosquito vector (Additional file 3), while in *Toxoplasma* the highest level of expression was associated with unsporulated oocysts.

### Assessment of dN/dS and in silico prediction of B cell epitopes of transmission-blocking candidate genes

**Fig. 4:**
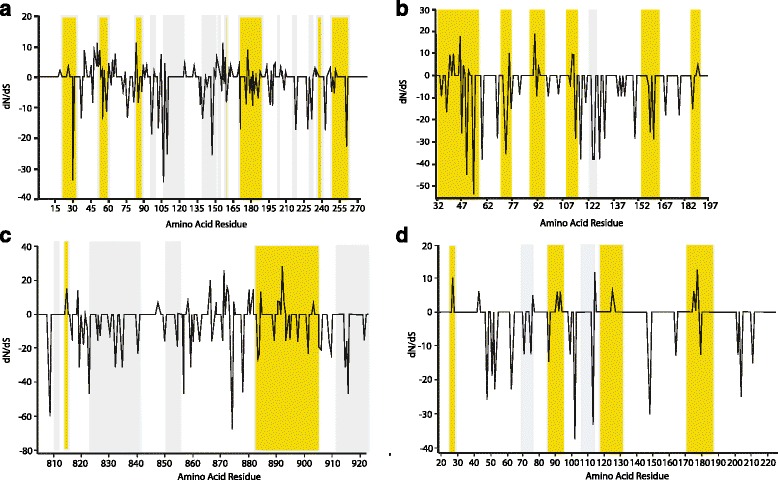
Diversifying selection and B cell epitopes in candidate genes. dN/dS computed from allelic sequences of (**a**) *TA17050*; (**b**) *TA10955*; (**c**) *TA03640* and (**d**) *TA20855*, plotted against predicted B cell epitope regions (>1 amino acid, shaded areas) from GeneDB reference sequences for each protein. Epitopes with evidence of positive selection (peaks above 0) are shaded *yellow*, epitopes with no evidence of positive selection are shaded *grey*. For *TA20855* and *TA10955*, but not *TA17050* or *TA03640*, there was a statistically significant association between predicted epitopes and positive dN/dS, reflected in the figure by more *yellow* shaded areas, relative to *grey*

Genes encoding antigens exposed to a protective immune response often display an elevated ratio of non-synonymous (dN) nucleotide substitution to synonymous substitution (dS) across allelic sequences [36, 37]. In contrast, genes encoding proteins specific to vector stages and not exposed to an acquired protective immune response may show more limited levels of selection for amino acid substitution [38]. To assess whether the putative proteins encoded by transmission-blocking candidate genes may be exposed to the immune response or act as hidden antigens, the level of dN/dS was computed for three candidate genes with evidence of elevated expression in tick stages. This was performed in comparison to the *Tams1* gene, as the level of dN/dS has been found to be relatively high among *Tams1* alleles [14, 16]. Allelic sequences were generated for all four selected genes from DNA representing a panel of parasite isolates: *T. annulata* Ankara (Turkey), Hissar (India), 9A (Tunisia) and UmBanein24 (Sudan). For each gene a minimum of 48 sequences were obtained and distinct consensus sequences selected. The dN/dS ratio was then computed as: 0.48 for *Tams1* (*TA17050*) with six significantly positive selection sites at *p* < 0.1; 0.13 for TA10955 with three significantly positive selection sites at *p* < 0.1; 0.19 for *TA03640* with no positively selected sites at *p* < 0.1; and 0.31 for *TA20855* with no significantly positively selected sites at *p* < 0.1. Thus, as expected, *Tams1* (*TA17050*) was shown to be the gene with strongest evidence for selection of amino acid substitutions. In contrast, the overall dN/dS ratio of *TA10955*, *TA03640* and *TA20855* was lower and few (*TA10955*) or no statistically significant positively selected sites were identified. However, visualisation of dN/dS plots (Fig. 4) revealed a degree of clustering of codons where dN/dS values were positive; this indicated that non-synonymous amino acid substitutions were tolerated, although there was insufficient power to determine these as statistically significant.

Amino acid substitutions that are positively selected to allow evasion from immune detection could be expected to coincide with the position of antigenic epitopes of *Theileria* proteins, as demonstrated for the T cell antigen gene, TA9 [37]. We used B-cell antibody epitope prediction software to identify regions of each protein where putative antibody epitopes could be detected. Using the Bepipred linear epitope prediction algorithm, individual amino acid residues were denoted as being within or outside predicted B-cell epitopes and this data superimposed onto the dN/dS plots. Preliminary inspection suggested that for at least some of the candidate genes, overlap exists between regions harbouring amino acid substitutions and predicted B cell epitopes (see Fig. 4). To test overlaps for statistical significance, every residue was classified for predicted epitope (yes or no) and evidence of positive selection (i.e. positive dN/dS values: yes or no) and a chi-square test performed. For candidate genes (*TA10955* and *TA20855*) there was a robust relationship between regions of amino acid substitution and regions of predicted epitopes: thus, sites with positive dN/dS scores, though rare, were significantly more likely to occur in regions of predicted epitopes than in non-epitope regions (*TA10955 X* ^2^ = 6.53 *p* = 0.011; *TA20855 n* = 243 *X* ^2^ = 938 *p* = 0.002). Taken together, the results suggest that these two candidate genes encode polypeptides with putative B-cell epitopes that exhibit weak, but detectable, evidence for selection of amino acid substitution.

## Discussion

The primary aim of this study was to use a bioinformatic approach to identify candidate genes encoding proteins with the potential to induce an immune response that could block transmission of *Theileria annulata* by the tick vector. Moreover, given that sexual reproduction is likely to have been retained by all vector-borne Apicomplexa [39], a secondary aim was to identify candidate genes that show a degree of conservation across *Theileria* species and related genera, particularly *Babesia*, as the two genera can be endemic over the same geographical region [40]. Two types of transmission-blocking candidate antigen were predicted: firstly (type 1), surface antigens required for the early phase of infection in the tick may be expected to be present in the bovine host and exposed to its acquired immune response, hence these antigens were expected to display a degree of antigenic diversity; secondly (type 2), surface proteins exclusive to stages present within the tick that perform an important biological function, such as gamete fertilisation, and may possess conserved epitopes that could induce a transmission-blocking antibody response if used as an antigen.

Based on results of our screen we identified twelve candidate genes, some of which possessed characteristics that allow placement into either type 1 or type 2 antigens. Thus, genes whose microarray expression level is elevated in the piroplasm but lower in the sporozoite stage are more likely to be expressed as proteins at the merozoite/piroplasm stage and may be present only in the initial phase of infection in the tick vector. This premise is supported by the observation that this group includes the genes, *TA17050* and *TA13810*, which encode the known major merozoite/piroplasm surface antigen, Tams1, and the 23 kDa piroplasm surface antigen. Expression in the tick was determined for *Tams1* where the RNA level was shown to fall within 2 days (at 28 °C post-detachment) and continued to fall over the remainder of the tick time-course. Thus, it can be predicted that synthesis of Tams1 protein (and by extrapolation possibly the 23 kDa piroplasm surface antigen) is significantly reduced (or absent) following generation of gamete forms (Day 6–10). This does not preclude a role for Tams1 as a transmission-blocking candidate, as piroplasms may persist for days within the tick and the protein may be relatively stable.

The results for *TA20855* show that it would be unwise to predict an expression profile for tick stages based solely on the available microarray data. * TA20855* shows a similar microarray profile to that of *Tams1* (*TA17050*) but we have shown by qRT-PCR analysis that peak expression of *TA20855* does not occur until around Day 10 post-detachment, a time-point associated with gamete fertilisation and production of zygotes [26]. The rapid fall in expression at the Day 15 time-point suggests a transient role prior to the production of kinetes, with a logical prediction being that the encoded protein is specific to gametes and perhaps performs a role in fertilisation or zygote development, although a role post Day 10 cannot be totally discounted if the protein is highly stable. BLAST analysis revealed a region of considerable identity, particularly over a predicted 8-cysteine structural domain, with genes encoding predicted membrane proteins in other Apicomplexa and in the chromerid, *Vitrella*. Homologues in *Plasmodium* show a transient peak in late-stage gametocytes (Additional file 3), while differential expression in *Toxoplasma* shows elevated expression associated with the unsporulated oocyst. The results suggest that this gene could be a remnant of the ancestral machinery of apicomplexan sexual reproduction. Based on the gene model it is likely to be an integral membrane protein, but with a significant proportion predicted to be extracellular. The region of greatest amino acid identity across homologues contains 8 spatially conserved cysteines and spans the region(s) predicted to act as a transmembrane helix. TM helices with conserved patterns of residues are unusual and indicate potential functional significance [41]. This is supported by evidence for conservation of amino acid substitution across *T. annulata* alleles in the region of the molecule predicted to be within the cell membrane or cytoplasm (see Fig. 4d). The function on the molecule can only be speculated upon at present, but the two most likely possibilities are as a ligand or a structural surface molecule that protects the parasite from the extracellular environment.

Genes that display an elevated level of RNA expression associated with the sporozoite stage may encode surface proteins whose function is primarily required after gamete fertilisation/zygote production. The gene (*TA03755*) encoding the major sporozoite surface antigen SPAG-1 [42] and a gene encoding a putative papain cysteine protease were placed in this category. *TA10955* was found to display peak expression at Day 15 of the tick time-course, indicating that the encoded protein may not be present until the later part of the life cycle in the tick. The predicted protein shows strongest similarity to the serine repeat antigen family (SERA) of *P. falciparum,* identified as important asexual blood-stage antigens (reviewed by [43]). The *Theileria* SERA represents a phylogenetic out-group to *Plasmodium* SERAs [32], with similarity over the peptidase domain of the predicted protein, but not the antigenic N-terminal domain identified for *Plasmodium* SERA5. Members of the *Plasmodium* SERA family function in merozoite egress, and have been implicated in sporozoite egress from the oocyst within the mosquito, providing a potential target for transmission blocking strategies [32, 44]. Based on its RNA expression pattern, the *Theileria* protein may function in a similar manner, promoting release of kinetes or sporozoites from infected tick cells. Whether this involves a surface associated location or secretion of the proteinase into the host cell environment would require validation.

The *TA03640* gene has an expression profile that is similar to *TA20855* but does not show a significant drop at Day 15. It is expressed at a higher level in the sporozoite relative to the merozoite/piroplasm, suggesting that production of the protein occurs within the tick. *TA03640* contains a pfs48_45 domain present in members of the 6-cys family in *Plasmodium*, including the gametocyte and gamete surface proteins Pfs48/45 and Pfs230 [33, 34]. Expression of a 6-cys encoding gene in *B. bovis* has been reported in merozoites [45]. However, a more recent study demonstrated low level expression in blood stages and elevated expression of at least seven out of ten *B. bovis* 6-cys genes by stages within the tick vector [46]. Thus, the elevated expression at the Day 10 and 15 time-points post-tick detachment suggests that *Theileria TA03460* may play a role in mediating transmission, as proposed for related vector-borne Apicomplexa. Based on demonstration that antibody responses generated against Plasmodium P48/45 and P230 can block transmission [34, 47] 6-cys domain surface antigens provide a target for development of transmission blocking vaccines against *Theileria* as well as *Babesia* [46].

The *in silico* analysis performed in this study indicated that the tested genes encode polypeptides with predicted B cell epitopes indicating that they have antigenic properties. In general, and relative to the merozoite/piroplasm major antigen gene *Tams1*, amino acid diversity is limited and no positively selected amino acid substitution sites were predicted for *TA03640* or *TA20855*. This could be taken as evidence that they may operate as “hidden antigens” and provide a target that shows conservation across species isolates, as proposed for the *Babesia* 6-cys candidate genes [46]. However, for *TA20855* (and *TA10955*) predicted epitopes coincided with certain positions where there is evidence for allelic amino acid substitution, and these were most evident in the region of the molecule modelled to be on the extracellular side of the cell membrane (i.e. N-terminal of residue 217). Evidence for selection of amino acid substitution has been reported for transmission-blocking proteins in *Plasmodium*, with the conclusion that this is driven by a protective immune response and/or mating interactions [34]. The former would require exposure to an acquired immune response, which has been shown to occur for *Plasmodium* transmission blocking antigens ([34] and references therein). Although our data indicates mRNA expression peaks in stages found within the tick, further work is required to assess whether polypeptides encoded by *TA10955*, *TA03640* and *TA20855* are hidden from or exposed to the immune response of the bovine host.

*Tams1* (*TA17050*) alleles showed the highest dN/dS ratio with six significant positive selection sites: however, areas of amino acid substitution did not show strong co-localisation with predicted B cell epitopes. One possible explanation is that divergent epitopes for this surface antigen are thought to be highly conformational dependent, are sensitive to mild periodate treatment and may not have been predicted by the algorithm. In addition, epitopes that are internal to the folded molecule and are not exposed to a protective immune response are likely [48]. Thus, to be fully effective as a vaccine candidate an antigen profile encompassing a wide range of divergent epitopes would need to be generated.

Based on the results of this study, at least two genes (*TA03640*, *TA20855*) demonstrate that transmission-blocking candidates can show a degree of conservation across related genera (*Babesia*, *Theileria*, *Plasmodium*). This implies that additional candidates may exist, as several other classes of protein are known to play a functional role in transmission of *Plasmodium*. Indeed, a preliminary screen for *Theileria* orthologues of *Plasmodium* transmission-blocking candidate genes and analysis (using criteria defined in methods) of the expression profile in *T. annulata* yields several other candidates (see Additional files 4, 5 and 6) including: a second *Theileria* 6-cys gene (*TA14250*); a gene (*TA09115*) with orthology to genes encoding the HAP2 protein that has been proposed to function as a gamete membrane fusogen in *Plasmodium* and many other protists [49–51]; a gene (*TA19820*) encoding a domain with orthology to the CPW-WPC domain encoded by surface proteins associated with *Plasmodium* transmission stages including the developing ookinete [52]. Further candidates are likely to be identified with a genome wide screen comparing bovine to tick stage transcriptome data.

## Conclusions

A bioinformatics screen has identified candidate genes encoding proteins with characteristics that allow prediction they have potential to block transmission of *Theileria* parasites. Given the economic loss associated with sub-clinical infection of *T. annulata* and the role of carrier animals in generating new clinical outbreaks, we believe further testing of candidates using a multi-antigen approach, possibly combined with an anti-tick component [53, 54], is warranted. Since a degree of conservation across vector borne Apicomplexa clearly exists for genes that promote transmission through the arthropod, it should be possible to consider development of generic strategies that are effective against this important group of pathogens. Progress towards this goal will depend on funded vaccine trials, these may be expedited by using smaller animal models to test candidates conserved across the Piroplasmida.

## Additional files

Additional file 1: Revised gene models for *T20855* and *TA19820* validated by RNA-seq reads, and alternate prediction of TM helices of *TA20855* using different software. (DOCX 236 kb)

Additional file 2: qRT-PCR and allelic sequencing primers (DOCX 16 kb)

Additional file 3: Transciptome data mined from EuPathDB for *Plasmodium* and *Toxoplasma* homologues of gene *TA20855*. (DOCX 50 kb)

Additional file 4: *TA14250* encodes a second 6-cys (s48_45) domain protein, predicted to be expressed in the tick vector. (DOCX 49 kb)

Additional file 5: *TA09115* encodes the HAP2 domain found in proteins essential for gamete fusion, predicted to be expressed in the tick vector. (DOCX 42 kb)

Additional file 6: *TA19820* encodes a CPW-WPC domain protein, predicted to be expressed in the tick vector. (DOCX 22 kb)

## Abbreviations

CDS: Coding DNA sequence
dN/dS: Ratio of non-synonomous (dN) to synonomous (dS) nucleotide substitutions
GPI: Glycosylphosphatidylinositol
TBD: Tick borne disease
TBV: Transmission blocking vaccine
TM: Transmembrane

## References

[1] Gharbi M, Sassi L, Dorchies P, Darghouth MA (2006). Infection of calves with *Theileria annulata* in Tunisia: Economic analysis and evaluation of the potential benefit of vaccination. Vet Parasitol.

[2] Gharbi M, Touay A, Khayeche M, Laarif J, Jedidi M, Sassi L, Darghouth MA (2011). Ranking control options for tropical theileriosis in at-risk dairy cattle in Tunisia, using benefit-cost analysis. Revue Scientifique et Technique-OIE.

[3] Rasulov I, Fish L, Shkap V (2008). Vaccination of cattle against tropical theileriosis in Uzbekistan using autochthonous live vaccine. Vaccine.

[4] Mhadhbi M, Naouach A, Boumiza A, Chaabani MF, Ben Abderazzak S, Darghouth MA (2010). In vivo evidence for the resistance of *Theileria annulata* to buparvaquone. Vet Parasitol.

[5] Benelli G, Pavela R, Canale A, Mehlhorn H. Tick repellents and acaricides of botanical origin: a green roadmap to control tick-borne diseases? Parasitol Res. 2016;115:2545–60. 10.1007/s00436-016-5095-1 27146901

[6] Graham OH, Hourrigan JL (1977). Review article: Eradication programs for the arthropod parasites of livestock. J Med Entomol.

[7] Jirapattharasate C, Moumouni PFA, Cao S, Iguchi A, Liu M, Wang G, Zhou M, Vudriko P, Changbunjong T, Sungpradit S (2016). Molecular epidemiology of bovine *Babesia* spp. and *Theileria orientalis* parasites in beef cattle from northern and northeastern Thailand. Parasitol Int.

[8] Sutton AJ, Karagenc T, Bakirci S, Sarali H, Pekel G, Medley GF (2012). Modelling the transmission dynamics of *Theileria annulat*a: model structure and validation for the Turkish context. Parasitol.

[9] George JE, Pound JM, Davey RB (2004). Chemical control of ticks on cattle and the resistance of these parasites to acaricides. Parasitol.

[10] Merino O, Alberdi P, Perez de la Lastra JM, de la Fuente J. Tick vaccines and the control of tick-borne pathogens. Front Cell Infect Microbiol. 2013;3:30. 10.3389/fcimb.2013.00030 PMC3705209 23847771

[11] Jeyabal L, Kumar B, Ray D, Azahahianambi P, Ghosh S (2012). Vaccine potential of recombinant antigens of *Theileria annulata* and *Hyalomma anatolicum anatolicum* against vector and parasite. Vet Parasitol.

[12] Boulter NR, Brown CG, Kirvar E, Glass E, Campbell J, Morzaria S, Nene V, Musoke A, d’Oliveira C, Gubbels MJ, Jongejan F, Hall FR (1998). Different vaccine strategies used to protect against *Theileria annulata*. Ann NY Acad Sci.

[13] Gubbels MJ, Katzer F, Shiels BR, Jongejan F (2001). Study of *Theileria annulata* population structure during bovine infection and following transmission to ticks. Parasitol.

[14] Gubbels MJ, Katzer F, Hide G, Jongejan F, Shiels BR (2000). Generation of a mosaic pattern of diversity in the major merozoite-piroplasm surface antigen of *Theileria annulata*. Mol Biochem Parasitol.

[15] Katzer F, Carrington M, Knight P, Williamson S, Tait A, Morrison IW, Hall R (1994). Polymorphism of *SPAG-1*, a candidate antigen for inclusion in a sub-unit vaccine against *Theileria annulata*. Mol Biochem Parasitol.

[16] Katzer F, McKellar S, Ben Miled L, d’Oliveira C, Shiels B (1998). Selection for antigenic diversity of *Tams1*, the major merozoite antigen of *Theileria annulata*. Ann NY Acad Sci.

[17] Shiels BR, d’Oliveira C, McKellar S, Ben Miled L, Kawazu S, Hide G (1995). Selection of diversity at putative glycosylation sites in the immunodominant merozoite/piroplasm surface antigen of *Theileria* parasites. Mol Biochem Parasitol.

[18] Carter R, Stowers A (2001). Current developments in malaria transmission-blocking vaccines. Expert Opin Biol Ther.

[19] Pradel G (2007). Proteins of the malaria parasite sexual stages: expression, function and potential for transmission blocking strategies. Parasitol.

[20] Kaslow DC. Transmission blocking vaccines. In: Hofman SL, editor. Malaria vaccine development. Washington: ASM Press; 1996. pp. 181–228.

[21] Carter R, Mendis KN, Miller LH, Molineaux L, Saul A (2000). Malaria transmission-blocking vaccines—how can their development be supported?. Nat Med.

[22] Carter R (2001). Transmission blocking malaria vaccines. Vaccine.

[23] Wu Y, Sinden RE, Churcher TS, Tsuboi T, Yusibov V (2015). Chapter Three-Development of Malaria Transmission-Blocking Vaccines: From Concept to Product. Adv Parasitol.

[24] Pieszko M, Weir W, Goodhead I, Kinnaird J, Shiels B. ApiAP2 Factors as Candidate Regulators of Stochastic Commitment to Merozoite Production in Theileria annulata. PLoS Negl Trop Dis. 2015;9:e0003933. 10.1371/journal.pntd.0003933 PMC4537280 26273826

[25] Langmead B, Salzberg SL. Fast gapped-read alignment with Bowtie 2. Nat meth. 2012;9:357–9. 10.1038/nmeth.1923 PMC3322381 22388286

[26] Mehlhorn H, Schein E (1984). The piroplasms: life cycle and sexual stages. Adv Parasitol.

[27] Walker AR, Fletcher JD, McKellar SB, Bell LJ, Brown CG (1985). The maintenance and survival of *Theileria annulata* in colonies of *Hyalomma anatolicum anatolicum*. Ann Trop Med Parasitol.

[28] Pond SL, Frost SD (2015). Datamonkey: rapid detection of selective pressure on individual sites of codon alignments. Bioinformatics.

[29] Pond SL, Frost SD (2015). Not so different after all: a comparison of methods for detecting amino acid sites under selection. Mol Biol Evol.

[30] Larsen JEP, Lund O, Nielsen M (2006). Improved method for predicting linear B-cell epitopes. Immunome Res.

[31] Sako Y, Asada M, Kubota S, Sugimoto C, Onuma M (1999). Molecular cloning and characterisation of 23-kDa piroplasm surface proteins of *Theileria sergenti* and *Theileria buffeli*. Int J Parasitol.

[32] Arisue N, Hirai M, Arai M, Matsuoka H, Horii T (2007). Phylogeny and evolution of the SERA multigene family in the genus *Plasmodium*. J Mol Evol.

[33] van Dijk MR, Janse CJ, Thompson J, Waters AP, Braks JAM, Dodemont HJ, Stunnenberg HG, van Gemert G-J, Sauerwein RW, Eling W (2001). A central role for P48/45 in malaria parasite male gamete fertility. Cell.

[34] Van Dijk MR, Van Schaijk BCL, Khan SM, Van Dooren MW, Ramesar J, Kaczanowski S, van Gemert G-J, Kroeze H, Stunnenberg HG, Eling WM (2010). Three members of the 6-cys protein family of *Plasmodium* play a role in gamete fertility. PLoS Pathog.

[35] Moore RB, Oborník M, Janouškovec J, Chrudimský T, Vancová M, Green DH, Wright SW, Davies NW, Bolch CJS, Heimann K (2008). A photosynthetic alveolate closely related to apicomplexan parasites. Nature.

[36] Endo T, Ikeo K, Gojobori T (1996). Large-scale search for genes on which positive selection may operate. Mol Biol Evol.

[37] MacHugh ND, Weir W, Burrells A, Lizundia R, Graham SP, Taracha EL, Shiels BR, Langsley G, Morrison WI (2011). Extensive polymorphism and evidence of immune selection in a highly dominant antigen recognised by bovine CD8 T cells specific for *Theileria annulata*. Infect Immun.

[38] Kang J-M, Ju H-L, Moon S-U, Cho P-Y, Bahk Y-Y, Sohn W-M, Park Y-K, Cha SH, Kim T-S, Na B-K (2013). Limited sequence polymorphisms of four transmission-blocking vaccine candidate antigens in *Plasmodium vivax* Korean isolates. Malar J.

[39] Weedall GD, Hall N (2015). Sexual reproduction and genetic exchange in parasitic protists. Parasitol.

[40] Simuunza M, Weir W, Courcier E, Tait A, Shiels B (2011). Epidemiological analysis of tick-borne diseases in Zambia. Vet Parasitol.

[41] Venancio TM, Aravind L (2010). CYSTM, a novel cysteine-rich transmembrane module with a role in stress tolerance across eukaryotes. Bioinformatics.

[42] Williamson S, Tait A, Brown D, Walker A, Beck P, Shiels B, Fletcher J, Hall R (1989). *Theileria annulata* sporozoite surface antigen expressed in *Escherichia coli* elicits neutralizing antibody. Proc Natl Acad Sci U S A.

[43] Palacpac NMQ, Arisue N, Tougan T, Ishii KJ, Horii T (2011). *Plasmodium falciparum* serine repeat antigen 5 (SE36) as a malaria vaccine candidate. Vaccine.

[44] Aly ASI, Matuschewski K (2005). A malarial cysteine protease is necessary for *Plasmodium* sporozoite egress from oocysts. J Exp Med.

[45] Silva MG, Ueti MW, Norimine J, Florin-Christensen M, Bastos RG, Goff WL, Brown WC, Oliva A, Suarez CE (2011). *Babesia bovis* expresses Bbo-6cys-E, a member of a novel gene family that is homologous to the 6-cys family of *Plasmodium*. Parasitol Int.

[46] Alzan HF, Lau AOT, Knowles DP, Herndon DR, Ueti MW, Scoles GA, Kappmeyer LS, Suarez CE (2016). Expression of 6-Cys Gene Superfamily Defines *Babesia bovis* Sexual Stage Development within *Rhipicephalus microplus*. PLoS One.

[47] Outchkourov NS, Roeffen W, Kaan A, Jansen J, Luty A, Schuiffel D, van Gemert GJ, van de Vegte-Bolmer M, Sauerwein RW, Stunnenberg HG (2008). Correctly folded Pfs48/45 protein of Plasmodium falciparum elicits malaria transmission-blocking immunity in mice. PNAS.

[48] Katzer F, McKellar S, Ferguson MA, d’Oliveira C, Shiels BR (2002). A role for tertiary structure in the generation of antigenic diversity and molecular association of the *Tams1* polypeptide in *Theileria annulata*. Mol Biochem Parasitol.

[49] Liu Y, Tewari R, Ning J, Blagborough AM, Garbom S, Pei J, Grishin NV, Steele RE, Sinden RE, Snell WJ (2008). The conserved plant sterility gene HAP2 functions after attachment of fusogenic membranes in *Chlamydomonas* and *Plasmodium* gametes. Genes Dev.

[50] Mori T, Hirai M, Kuroiwa T, Miyagishima SY. The functional domain of GCS1-based gamete fusion resides in the amino terminus in plant and parasite species. PloS One. 2010;5:e15957. 10.1371/journal.pone.0015957 PMC3013147 21209845

[51] Wong JL, Johnson MA (2010). Is HAP2-GCS1 an ancestral gamete fusogen?. Trends Cell Biol.

[52] Kangwanrangsan N, Tachibana M, Jenwithisuk R, Tsuboi T, Riengrojpitak S, Torii M, Ishino T (2013). A member of the CPW-WPC protein family is expressed in and localized to the surface of developing ookinetes. Malar J.

[53] Ghosh S, Ray DD, Vanlahmuaka, Das G, Singh NK, Sharma JK, Azhahianambi P (2008). Progress in development of vaccine against *Hyalomma anatolicum anatolicum* -Indian scenario. Vaccine.

[54] Shakya M, Kumar B, Nagar G, de la Fuente J, Ghosh S (2014). Subolesin: A candidate vaccine antigen for the control of cattle tick infestations in Indian situation. Vaccine.

