# Supplementary material for: Identification of candidate transmission-blocking antigen genes in Theileria annulata and related vector-borne apicomplexan parasites
Source: BMC Genomics. 2017 Jun 5;18:438. doi: 10.1186/s12864-017-3788-1 (PMC5460460; doi:10.1186/s12864-017-3788-1)
Supplement: Supplementary file 1 — Revised gene models for T20855 and TA19820 validated by RNA-seq reads, and alternate prediction of TM helices of TA20855 using different software. (DOCX 236 kb) [file 12864_2017_3788_MOESM1_ESM.docx]

**Additional File 1. Revised gene models for *TA20855* and *TA19820* validated by RNA-seq reads, and alternate prediction of TM helices of *TA20855* using different software**.

**A**


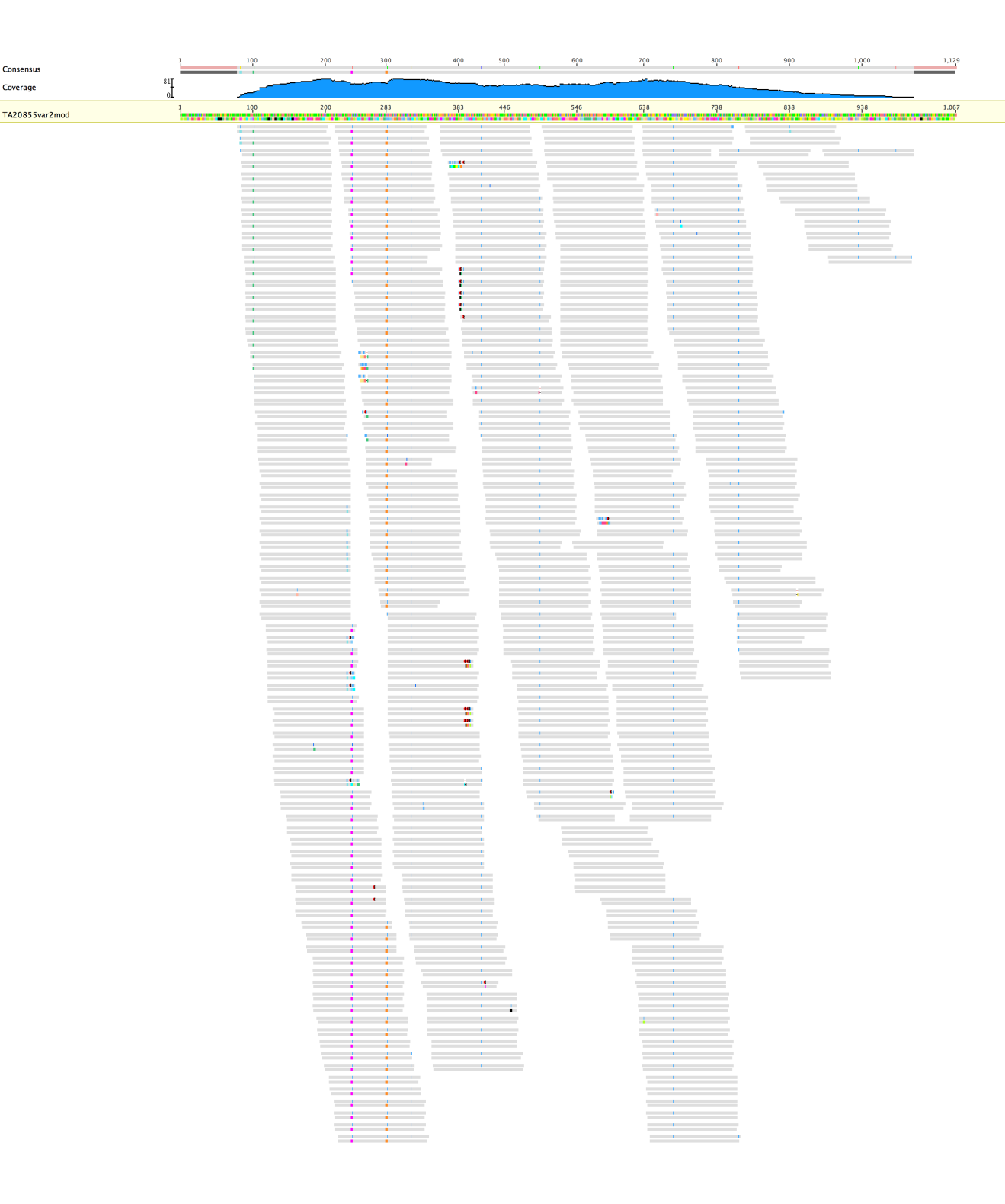


**B**


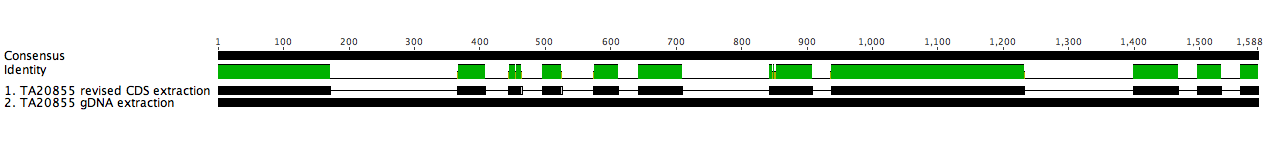


**C**


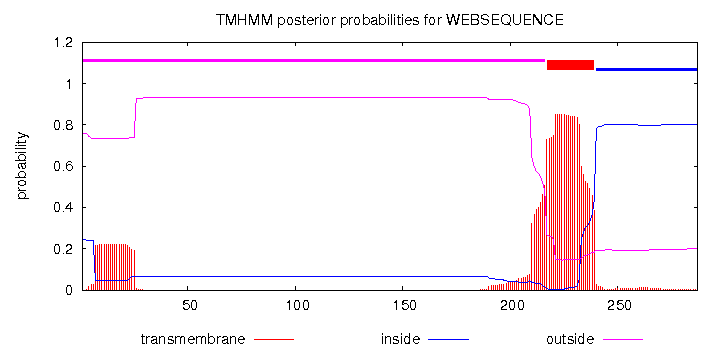

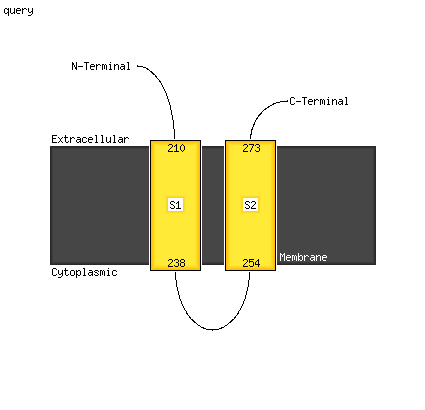


**D**

**
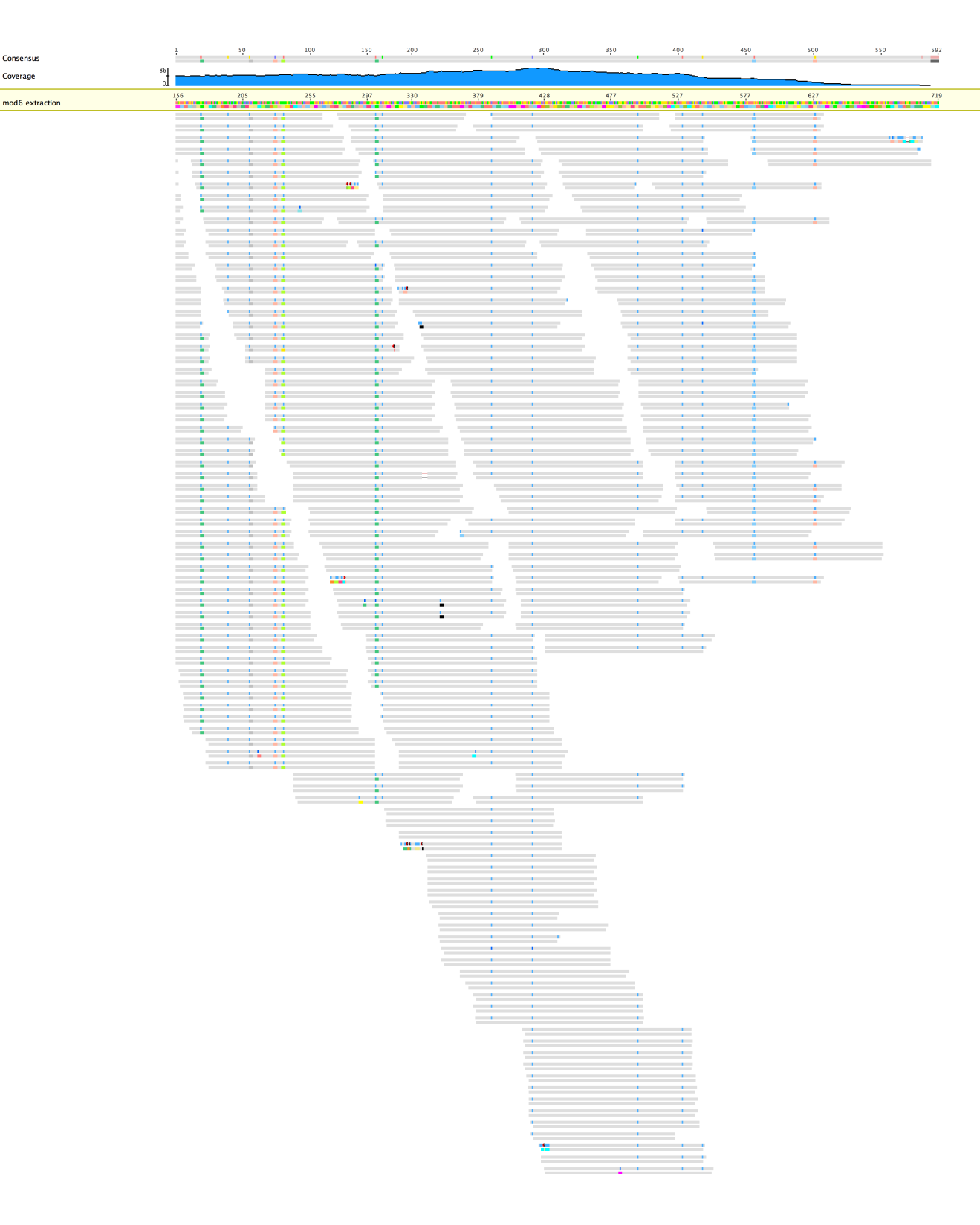
**

E


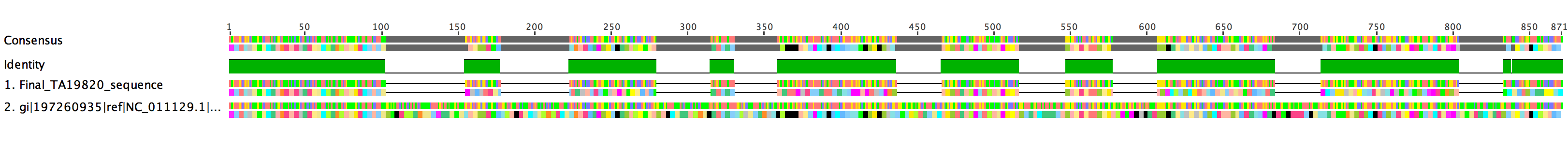


**A)** RNA-seq reads mapped against the revised CDS of *TA20855* (including UTRs) demonstrating complete coverage or predicted amino acid sequence: analysis of RNA-seq reads indicated a number of variant transcripts where intron 3 was not spliced out resulting in a stop codon, and termination of the protein after amino acid residue 78 (R). Such premature termination caused by splicing events has been proposed as a mechanism for control of gene expression in plants (Filichkin et al. 2010). **B)** Revised *TA20855* gene model aligned against gDNA sequence in GeneDB, highlighting the position of 11 exons. **C)** Prediction of one TM helix for TA20855 by TMHMM Server v2.0 and two helices by Memsat_SVM in Phyre^2^ (Kelly et al., 2015), the overall topology (majority of protein to the extracellular side of the membrane) is predicted to be similar by both algorithms. **D)** RNA-seq reads mapped against the revised CDS of *TA19820* (excluding UTRs) demonstrating complete coverage or predicted amino acid sequence. **E)** Revised *TA19820* gene model aligned against gDNA sequence in GeneDB, highlighting the position of 10 exons.
