# Supplementary material for: Identification of candidate transmission-blocking antigen genes in Theileria annulata and related vector-borne apicomplexan parasites
Source: BMC Genomics. 2017 Jun 5;18:438. doi: 10.1186/s12864-017-3788-1 (PMC5460460; doi:10.1186/s12864-017-3788-1)
Supplement: Supplementary file 2 — qRT-PCR and allelic sequencing primers (DOCX 16 kb) [file 12864_2017_3788_MOESM2_ESM.docx]

Additional File 2. qRT-PCR and allelic sequencing primers

Table 1: List of forward (F) and reverse (R) primers designed for qRT-PCR including melting temperature (Tm) and number of base pairs (bp) constituting the amplicon.

| **Gene Identification**  **Product Name** | **Primers** | **Tm (°C)** | **Amplicon length (bp)** |
| --- | --- | --- | --- |
| TA10955q  Papain-family cysteine protease | F: GGATAAACCAGAAGCCGAAAATAGA  R:CCCCTTCATACTGATAACCACACTA | 59 | 208 |
| TA17050q  Merozoite-piroplasm surface antigen Tams1 | F: AGGACCACCCTCAAGTTCTTATATT  R: GTAGCCATCCGCGACAGTTA | 59 | 189 |
| TA03640q  6-cysteine protein | F: TGGATGCGGAGTGAATGGAC  R: AAACATCCCGGAGGGTCAAC | 59 | 100 |
| TA20855q  Conserved hypothetical protein | F: CAATTCAACAGAAATCGCACAT  R: CTTTCTCGCCATATTTCTCAC | 59 | 150 |
| TA10720q  Heat shock protein 90 | F: CTAGGAACAATAGCAGAATCAGGAAC  R: AGTAAAATCCAACTCCGAACTGTC | 59 | 100 |
| TA11610  Heat shock protein 70 | F: ACGCAAATGGAATCCTCAAC  R: TATTCGTCGTGCTCTGCTAA | 52 | 334 |

Table 2: List of forward (F) and reverse (R) primers specifically designed to amplify the entire length, or large fragments, of three candidate genes (melting temperature (Tm) and number of base pairs (bp).

| **Gene Identification**  **Product Name** | **Primers** | **Tm (°C)** | **Amplicon length (bp)** |
| --- | --- | --- | --- |
| TA10955 | F: GCCAACCATCCTGGATATGA  R: TCATGCAGTCGTAGTCTTTGTCA | 52 | 1609 |
| TA17050  Merozoite-piroplasm surface antigen Tams1 | F: TTGTCCAGGACCACCCTCAAG  R: ACGATGAGTACTGAGGCGAAGA | 55 | 815 |
| TA03640 (Fragment 1)  TA03640 (Fragment 2: used for sequencing) | F: GCTGACGGTCTACAAGAGCT  R: TAGCATGTCGCAGTACGGAT  F: AATCCGTACTGCGACATGCTATT  R: CGACATCATAATTCCCTTCACCG | 59  59 | 1437  1956 |
| TA20855 | F: GTTAGGTTGTGTAAAGTTGATGTTG  R: ACCTCCATAAATACGGCACAA | 7 | 1533 |
