# Supplementary material for: Identification of candidate transmission-blocking antigen genes in Theileria annulata and related vector-borne apicomplexan parasites
Source: BMC Genomics. 2017 Jun 5;18:438. doi: 10.1186/s12864-017-3788-1 (PMC5460460; doi:10.1186/s12864-017-3788-1)
Supplement: Supplementary file 3 — Transciptome data mined from EuPathDB for Plasmodium and Toxoplasma homologues of gene TA20855. (DOCX 50 kb) [file 12864_2017_3788_MOESM3_ESM.docx]

**Additional File 3. Transcriptome data mined from EuPathDB for *Plasmodium* and *Toxoplasma* homologues of gene *TA20855*.**

**A**


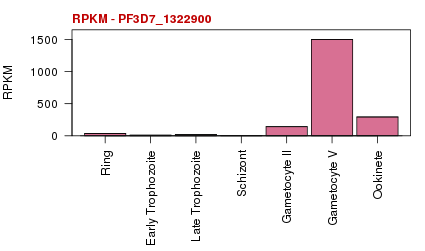


**B**

|  |
| --- |


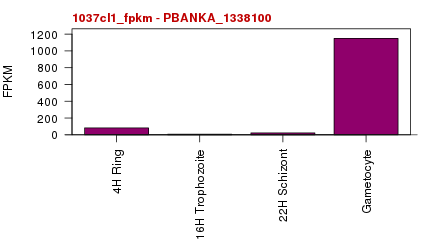


**C**


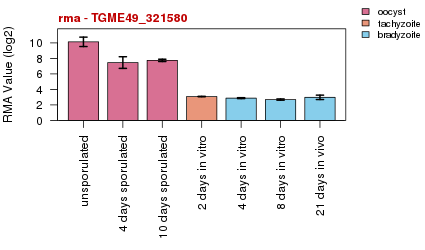


**A)** Expression profile of *PF3D7_1322900* generated from Illumina-based sequencing of *P.falciparum* 3D7 mRNA representing two gametocyte stages (II and V), the ookinete, and four time points of erythrocytic stages - ring, early trophozoite, late trophozoite, and schizont (<http://plasmodb.org/plasmo/app/record/gene/PF3D7_1322900>): **x-axis,** life cycle stages cycle: **y-axis,** reads per kilobase of exon model per million mapped reads (RPKM). **B)** Expression profile of *PBANKA_1338100* generated from Illumina-based sequencing of RNA from five different stages of *P. berghei* ANKA development (4h Ring, 16h Trophozoite, 22h Schizont, Gametocytes (<http://plasmodb.org/plasmo/app/record/gene/PBANKA_1338100>): **x-axis,** stages of *P.berghei* ANKA development; **y-axis,** reads per kilobase of exon model per million mapped reads (RPKM). **C)** Transcriptome analysis for *TGME49_321580* of oocyst, tachyzoite and bradyzoite development in the type II strain M4 of *T. gondii* (<http://toxodb.org/toxo/app/record/gene/TGME49_321580>): X-axis, 1. time post infection or sporulation (i.e. 4d vs 8d bradyzoites and 4d vs 10d sporulated oocyst), 2. developmental form (oocyst, tachyzoite, bradyzoite), 3. bradyzoite samples - obtained *in vitro* vs. *in vivo*: Y-axis, RMA Normalized Values (log base 2).
