# Supplementary material for: Identification of candidate transmission-blocking antigen genes in Theileria annulata and related vector-borne apicomplexan parasites
Source: BMC Genomics. 2017 Jun 5;18:438. doi: 10.1186/s12864-017-3788-1 (PMC5460460; doi:10.1186/s12864-017-3788-1)
Supplement: Supplementary file 4 — TA14250 encodes a second 6-cys (s48_45) domain protein, predicted to be expressed in the tick vector. (DOCX 49 kb) [file 12864_2017_3788_MOESM4_ESM.docx]

**Additional file 4. *TA14250* encodes a second 6-cys (s48_45) domain protein, predicted to be expressed in the tick vector.**

**A**

T orientalis MKLINSVVTFLFISLVRFASSSSFDFSSIGEAGLSAVKKNVAKATQFALNYTVFNKTLTF 60

T annulata *MQLLKSTLFIYFIIYSG*-----*SFC*---*SAKFGLDFL*ARKMNSAVDLAMNYAIMNETLSF 52

T parva *MAMYKSRLFIYFIIYSC*-----*TFC*---*HAKYGFKYF*ANKVNNAVNLAINYAVLNETLSF 52

* : :* : : ** :* .: *:. . .:: .*.::*:**:::*:**:*

T orientalis KDINDFNSGEDNVLRYTLPPGSALKVFCGNEHTRKSGLITLYPPDLTTHTLAPMSKGGIQ 120

T annulata KEAADFTADGESFMSYTLEPGSAIKFYCGTSQTIDSRQILMYPQDLKTNSLAPMSKSGIN 112

T parva KEPGDFTAEGESFKSYTLEKGGAIKFYCGNNETEEGRKIKMYPEDLKKTSLAPMSKSGIT 112

*: **.: :.. *** *.*:*.:**...* .. * :** **.. :******.**

T orientalis DSLGRVMKNFDVFRSDRTLVHSAPEWFGGSYMFQHPYSSTIVSRDPEFTLNFACVYKLLS 180

T annulata HSIKRVVRNFDLFRSDSTLIYALAEFLGGGYMIQYPADSVIVSNNPDFSLNFACVYDPNA 172

T parva HSIKRVINNFGLFRSDSTLIYSLSEFLGGGYLIQYPADAVIVSSDPNFSLNFACVYDPNY 172

.*: **:.**.:**** **::: *::**.*::*:* .:.*** :*:*:*******.

T orientalis ED--ASAASEAGESAQKDVLYRWVEIKFTDVLPISYGCGTGNYPLFSNLSAVKNLLSPNG 238

T annulata PYTDNEPDELNGGEEKKDLLYRWVEVKFKNVYPMSYGCGSKGYPLFSNLSPGYGLDFLSE 232

T parva DTSGENKSDELNTEEKKDLLYRWIEVKFTDVLPISYGCGSGNHPLFNNLSPENGPDKLEG 232

. . . . :**:****:*:**.:* *:*****: .:***.*** . .

T orientalis PPQQTSTMCEIEPEPGMIVGLYCADDQTFDDKSCFVDRKKMTYYDPEFKHTNRPGRLHLY 298

T annulata TFLQPGSNCEIEPKPGMVIGIYCAVGETFNDELCFRDASGNIFFDQNYKHSG-GLHLHLY 291

T parva TFLQTRAFCEIEPKPGMVIGIYCAAGETFNDEFCFRDSSGKTYYDPDFKYDGAGQRLHLF 292

* : *****:***::*:*** .:**:*: ** * . ::* ::*: . :***:

T orientalis KIPDTGLVTDLPFSCKCRDEKGNVTSELKVVKRTEHLCDFTRVFENLRPRQTIPLQLCRH 358

T annulata RVPDSGYDSDVNFLCECKGKNGETRAQVRVRKYSTMNCDLTKIFSNYKPGKKITLQACRR 351

T parva RVPENGFGGDVYFLCECRGKNKDTKAQVKIIKASVLSCDLTKIFEKHEPGKKISLQACRR 352

::*:.* *: * *:*:.:: :. ::::: * : **:*::*.: .* :.* ** **:

T orientalis NLHPGESLRLIAPPNNSVVNMMGLKRFSIIEPTELRYLTYIQALGSRRDVLANLRDIMIT 418

T annulata DLRPGESLKLTVPANFSKFNYMNASYSSLLEPIEVRFYAYRQKLDDYQVRKVKLRDLIVG 411

T parva DLRPGESLKVTVPKNYTEFYYFKHKYSSLFEPINVQHYAYRQKLDDNEVRKVKLSDLIVG 412

:*:*****:: .* * : . : . *::** :::. :* * *.. . .:* *:::

T orientalis TGFSVEKVHATDRLEYVFKYEKDAILVQTTEVTGLNYYWTLMNSPNADRQHVKSPSQEGH 478

T annulata NGLDIEKSVVPGGVQYIFKYKKDSILILTSETVSFEYFWTLFYKNPRERDVISYLNMNEK 471

T parva DGLTIEKTPVRGAVEYLFKYNRDSILILTSETVSFEYFWTLFHRNPREREVISYLSLNEK 472

*: :** . . ::*:***::*:**: *:*...::*:***: :*: :. . : :

T orientalis INGVINIGLVPTDPYTIGCGTRNEFFIHEFVDFETSSENDKVISKCTVDANKGPIGFYCP 538

T annulata VVAVVSIGLFPTDPYTYGCGTRNDIFRKELLDFRSELINNKPASICRVDGKNSPIGFYCP 531

T parva VLAVVSIGLFPTDPYTYGCGVRNDFFRRELLDFRSEEINNKPTSICKVDGRNSPIGFYCP 532

: .*:.***.****** ***.**::* :*::**.:. *:* * * **..:.*******

T orientalis SPYLLEPRGCFNSVIVERTGEDGETTTHVRPLKSVLKNARASRSSHLVLLEVYKNFTRRP 598

T annulata KPFLLEPRDCFKSVLVSTDEG--EKS---VDLVKVDKHARHFTTKNLVVLDTYSNGGHVG 586

T parva RPFLLEPRDCFRSVLIQTDEG--EES---VDLVKVAKYARHFTTKHLVILDTYTTGKHEG 587

*:*****.**.**::. * : * .* * ** :.:**:*:.*.. :

T orientalis GDESDDESDDEDSRHSSFGSDFDALVCKCVDVRGNVMSTIKVLNPNKEIPES---LRKDY 655

T annulata PAQNEGGANYED---DNEYVNASEIMCKCVDEEGNLVASITVQLDKPVEAEKEGAEEDGG 643

T parva ---------------TNEYINTSEIMCKCVDEEGNLVASITVQLDKDTRADVGESSDSDN 632

. : . ::***** .**::::*.* : : ..

T orientalis E----------ERTKKSLKTIFNLTKVH 673

T annulata IPEPQENSGVEEDTGIPETTIY------ 665

T parva EEGSGEVGGNSDDDFRIETRIS------ 654

: . *

**B**


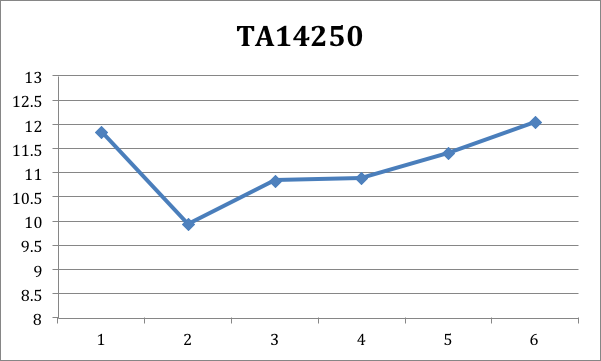


**A)** Alignment of 6-cys protein homologues of *T. annulata* (TA14250), *T. parva* (TP02_0629) and *T. orientalis* (TOT_020000614). Predicted signal peptides are shown in italics and s48_45 domains are highlighted in yellow; TA14250 1^st^ Pfam: s48_45=0.001, 2^nd^ Pfam: s48_45=2.2e-12. **B)** Microarray profile analysis predicts expression by stages within the infected tick. X-axis stages: 1 sporozoite; 2 macroschizont; 3 merozoite, day 4; 4 merozoite, day 7; 5, merozoite, day 9; 6 piroplasm. Y-axis: expression level expressed as log_2._
