# Supplementary material for: Identification of candidate transmission-blocking antigen genes in Theileria annulata and related vector-borne apicomplexan parasites
Source: BMC Genomics. 2017 Jun 5;18:438. doi: 10.1186/s12864-017-3788-1 (PMC5460460; doi:10.1186/s12864-017-3788-1)
Supplement: Supplementary file 5 — TA09115 encodes the HAP2 domain found in proteins essential for gamete fusion, predicted to be expressed in the tick vector. (DOCX 42 kb) [file 12864_2017_3788_MOESM5_ESM.docx]

**Additional file 5. *TA09115* encodes the HAP2 domain found in proteins essential for gamete fusion, predicted to be expressed in the tick vector.**

**A**

T.orientalis *MTSCYLNKGLLSALIKLCVFYTFLSIGG*-*FVKINVF*NDNSLFAEALVTGNVTVCVKNSKA 59

T.parva *MSSLGPFRSVFTSLIYFSILH*-*ILGFTSLFNFY*TTDSTGFFFVDSAVTGNITQCVRNSDK 59

T.annulata *MASIGVFRSLRASLVYITILYIILGPSSIFNFHNI*FNNRFFFVDSAVTGNITQCVRNSER 60

*:* :.: ::*: : ::: :*. . * . . :*.:: ****:* **:**.

T.orientalis LFDEKTCKNRLHTNIDLFHGDKYYHYIFRRKDDPTKGLYIIARTSNTSVRYRLDYKISVP 119

T.parva LFDDQTCVQRLHTNVDVSHGLREYHYIYRRKDDLSKGLYLVLKTSNTSLLYTLNYQTMVP 119

T.annulata LFDEKSCVMRLHTNVDVSHGLREFHYIYRRKDDLSKGLYLILKTSNTSVAYTLDYQTTVP 120

***:::* *****:*: ** : :***:***** :****:: :*****: * *:*: **

T.orientalis YFYREYTEGKTYGEVSYDPESFCQIGFHSNCTTSAKLPPGFATIPSFCCICDINVDGETN 179

T.parva LYYTDHTERWTYSEISGELKTSCKSVQNSKCTKKTQVPPGIDFLPRVCCICGLNVHKPTP 179

T.annulata YYYTDHTENWTSSEISGELRTSCKRVQNSKCTKTADVPPGIDFLPSVCCICGVNVHKSTP 180

:* ::** * .*:* : .: *: :*:**..:.:***: :* .****.:**. *

T.orientalis RARYNCDRH---RDSIAMTYSCLEVMEPWYNLYMMSYPPDLLRNTVFSIYKFDKSNGIIP 236

T.parva RADFKCGGFLAMGGRTALSMSCLEISEPWYKLYKTSYPPAISRSVTVNIYKFDSSTGIIP 239

T.annulata RANFRCSGF---GRRTVLSMSCLEAGAPWYKLYKTSYPPLVSRSVTVNIYKFDSSKGIMP 237

** :.*. . .:: **** ***:** **** : *.....*****.*.**:*

T.orientalis DLNLDKDGYFDNYDFKNRKNLDPSFKAEKDKVRPE----------VKRTSEHNKIVANYR 286

T.parva DVTLEDEDKFDNYDFKKREKKDPVIKSPEIKSR----------STKEITGKKDELHPNFR 289

T.annulata DVSLEDEGKFDNYDFKKRQKKDPVLKTRDLRSTSRPQPLDETYSSKELVKKEDELHPNFR 297

*:.*:.:. *******:*:: ** :*: . : : . :.::: *:*

T.orientalis NIIIDSTSKEVA-------------------------------IDDLDITISLLSSNTKN 315

T.parva RIIIDDTVKEEH-------------------------------INDLDVKITLLSSNTKD 318

T.annulata RIIIDDNVKEEKIGIKSLYSYSDNQYSNIPIFQYSQYTNINYCIDDLDVTISLLSSNTKD 357

.****.. ** *:***:.*:*******:

T.orientalis GSAPPMLDKYVAVPSFPRTNETVKGSSLIDKCMDGTWPKSLKCPKYMPQKICNYWRCTLN 375

T.parva GSAPPLFDKYVAIPSFPRTNETVKGSSLMDKCQDSTWKTKPECPKYMNPSLCDIWRCTLN 378

T.annulata GSAPPLFENYVAIPSFPRTNETVKGSSLMDKCEDSTWSKKPECPKxMNPSLCNIWRCTLN 417

*****::::***:***************:*** *.** .. :*** * .:*: ******

T.orientalis MRTIERDAVDTGGSQCNKIGYSYTAHDDHWNLCQMRASSCINRQLKWYLDEKKDQAKMPN 435

T.parva MRTVKMSAVDTDGLMCDKIGLSMKRWANQEEICNSSPGSCLKNQLKHYFDQEKDEAKLPK 438

T.annulata MRTVKKSAVDPSGMTCDKIGLTMRRWANQEEVCSTRPGTCLKNQMKWYLEQEKDDAKLPK 477

***:: .*** .* *:*** : :: ::*. .:*::.*:* *::::**:**:*:

T.orientalis FYGVEPAITIDTTQVPRSEDPEKKRTTWFQEDRIHYINYVHSEDDVSRYKIDTFEASITE 495

T.parva LYGVEPTFTAVKKDLSLPAVKEANKTTLDDPNRIHTLTYIHSKDDVTRLKIDTFDATVTE 498

T.annulata LYGVEPTFTAVKRDLSLPAIKEPNKTTLDDPNRIHTLTYIHSNDDVTRLKIDTFDATVTE 537

:*****::* . :: * ::** : :*** :.*:**:***:* *****:*::**

T.orientalis IIADFPGFILSTKIDKECKLNSPDICTLQVDVKNMGTFKLMIYLTGVNTSHFTINALCYA 555

T.parva IISDFPGFIVSAKMDGECEVSSEKGCNMELDVKNMGKFTHKNSILGVKKSEFTVRANCYD 558

T.annulata IISDFPGFIVSAKIDGECEVSSSKKCNMELDVKNMGKFIRFNNILGVKKSDYTIRATCFD 597

**:******:*:*:* **::.* . *.:::******.* : **:.*.:*:.* *:

T.orientalis DETSKAKENVIAEIDETTLNIKGNTNKLFNIPIKLSGPLSSEKSYCFVNLLSGTKKHLDA 615

T.parva DPDL---KNEVAQISETTLSIDGNKNKTVSIPIKLTGSLASEKGYCNIILLSGKKEMLDG 615

T.annulata DPIR---RNPVATISETVISIDANKNKTTSIPIKLTGSVSTQKGYCDIILLSGKKDTLDS 654

* .* :* *.**.:.*..*.** .*****:* ::::*.** : ****.*. **.

T.orientalis ATTSIKIKKVKPVVGMDPKYINPDVHTVS-----INVVPKQGYVEKSPPEPSEKKSSSTT 670

T.parva MKMEIKVKVKKETFGKDPVKVQ-DIVAAPSPKDKLTTPQVI--NPIVINQPGSKNDTKKE 672

T.annulata VKIDIKVKVKEKPIGADPQHRAQNVVGERNPQHKITIQPTQ---QHDGKDSTTKKTDKKD 711

. .**:* : .* ** :: :. : *: ..

T.orientalis KEKECTCATWNIFCMLFNFKTCVIAKVSKVFTYIMIGLG*IFLFIILLPVIIPLFGGLINL* 730

T.parva EESQCKCASWNIFCMLINFKICVSSYVSK*VLFYVLIALGILLLLILLPVLIP*LIVSLFKA 732

T.annulata DEPKCRCASWNIFCMLINFNLCISSYVSKVLFYVL*IALGILLLLILLPVLIPLFVTVF*KG 771

.* :* **:*******:**: *: : ****: *::*.***:*::*****:***: :::

T.orientalis *IV*KLCKMPYNAMERRRLEKMNTKVSYVNPADMRKEDLNDMKLAENTASLPGINVNLNLSD 790

T.parva LAGLIKTPLEALEQRRLKKKNNTQLEV--------------------------------- 759

T.annulata IAVLVKMPFEAIEQKRRNKRMLDNTNMTLDDF---------------------------- 803

:. * * * :*:*::* :* :

T.orientalis VKEQSRRYKPYEDPNISVQGQSLVESIESIASIHNKNGKSGHDQL 835

T.parva --------------------------------------------- 759

T.annulata --------------------------------------------- 803

**B**


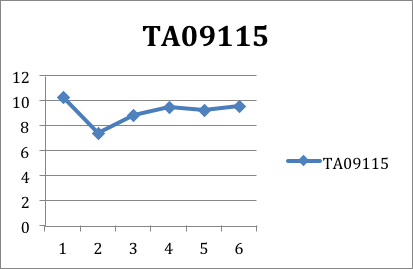


**A)** Alignment of HAP2 domain proteins of *T. annulata* (TA09115), *T. parva* (TP04_0574) and *T. orientalis* (TOT_040000351). Predicted signal peptides and transmembrane domains are shown in italics and HAP2 domains are highlighted in yellow; TA09115 Pfam HAP2-GCS1=1.6e-17. **B)** Microarray profile predicts expression by stages within the infected tick. X-axis stages: 1 sporozoite; 2 macroschizont; 3 merozoite, day 4; 4 merozoite, day 7; 5 merozoite, day 9; 6 piroplasm. Y-axis: expression level expressed as log_2._
