# Supplementary material for: Identification of candidate transmission-blocking antigen genes in Theileria annulata and related vector-borne apicomplexan parasites
Source: BMC Genomics. 2017 Jun 5;18:438. doi: 10.1186/s12864-017-3788-1 (PMC5460460; doi:10.1186/s12864-017-3788-1)
Supplement: Supplementary file 6 — TA19820 encodes a CPW-WPC domain protein, predicted to be expressed in the tick vector. (DOCX 22 kb) [file 12864_2017_3788_MOESM6_ESM.docx]

**Additional file 6. *TA19820* encodes a CPW-WPC domain protein, predicted to be expressed in the tick vector.**

P.vivax ------*MRQPCLLFLSLFVFSFFNFSECML*PWTKKRKAVNQMGIIKDMSQELRTKAEQLP 54

P.falciparum ------*MNYLSSFFCLILFLLYLNFCACMW*PWTKKWKAENQMAIIKDMSKEIRHKAETLP 54

T.parva *FNIMSITTDISSFYIVFIATFLTHSSYS*L----NKSDFESQTDIFKDLASKLKMASESVP 56

TA19820_revised *MKDRAFVTNIWHLSLAILTFNYIHYSFC*L----NKSKFEAQADLFKDIASKLKVASESVP 56

B.bovis ------*MLHYTNVVTILVLISHIAKGAR*I----RKNYTDTQLDLFKDIAKNIKQESLMMP 50

B.bigemina ------*MTSLVFIVATILGCVSQTDGSR*I----RKSYTDTQLDLFKDIAKNLKEESEHIP 50

. :. : .* : ::**: .::: : :*

P.vivax TPEDISAKIHRVDKEVIDKLNKDIIEEENLDKHKPHVCQEPAYERDYSYLCPEDWVKNSN 114

P.falciparum TPRDITNKIHRIDKDIIDQLNKDIIDEENLSKHKAHICLEPNYERDYKYLCPEGWIKNKN 114

T.parva TQADVIAKIKSIDSEEMRKLDEKIKREESEAVKDHGSCGDVSYVRDYSFECPEGWVLTGD 116

TA19820_revised TQSQVIDKIKSIDDEALKKIDDKIRKEESEDAKEHGSCGDISYERDYSFECPSGWTLTDD 116

B.bovis TSAEVIEKMKRIDEAEYKKIDKRIEKETAELTADHGSCGTVNYKRDYTHPCPEGWTPKSD 110

B.bigemina TTAQVVENMKRLDDAEFKRLDERIAKETAQLSEEHSSCGTVNYERDYSHACPSEWTLQAD 110

* :: ::: :* :::. * * . * * ***.. **. * :

P.vivax DQCWGIDYDGHCEALKYFQDYSVEEKKEFEMNCCVLWPKLKNEGMKG--AHKKDLLRGSI 172

P.falciparum GQCWGLHYDGHCESLKYFQEYNDNEKKEFELSCCVLWPKLKSDNKKK--SKKRKTIRGSI 172

T.parva GSCWGVNYGGNCASKQSFKWFSVGQKKDIEDKCCALWPRKLTVKEAGIKARKLHLVHGSV 176

TA19820_revised GTCWGMNYKGNCDSRQSFKWFSVDQKRDIENKCCAFWPRKLTAREAVSRVGKMELVHGSV 176

B.bovis GSCWGQGYKGPCEALQTFKWFTEEEKRSFEQRCCAFWPPVNLESISTSAKMLPTPLNGSV 170

B.bigemina GSCWGEHYTGPCEAIQTFKLFTKADKTKFEHRCCAFWPARGSRKRSVSTSALIGGLHGIV 170

. *** * * * : : *: :. :* .:* **.:** :.* :

P.vivax SSNNGLIIKPKYL 185

P.falciparum KSSNGLIIRPKNI 185

T.parva NFPDGRIIPPRG- 188

TA19820_revised NFQDGKIIPPRN- 188

B.bovis DHDNGMVIAARI- 182

B.bigemina DPHDGHIVPPRQ- 182

. :* :: :

**A)** Alignment of CPW-WPC domain proteins of *T. annulata* (TA19820), *T. parva* (TP01_0047), *P vivax* (PVP01_1125200), *P falciparum* (PF3D7_0624300), *B bigemina* (BBBond_0300360) and *B bovis* (BBOV_I000840) Predicted signal peptides and transmembrane domains are shown in italics and CPW-WPC domains are highlighted in yellow; TA19820 Pfam: PF3D7_0624300.1. The CPW-WPC domain is about 61 residues in length and has six well-conserved cysteine residues and six well-conserved aromatic sites. The *Theileria* gene models were based on a revised gene model for *TA19820* (indicated by divergence from homologues of related genera) that was validated by RNA-seq data (see Figures1). The gene models for *T. parva* and *T. orientalis* were predicted based on identification of exon and intron structure identical to that predicted for the *T. annulata* gene. **B)** The micro array profile predicts expression by stages within the infected tick. X-axis stages: 1 sporozoite; 2 macroschizont; 3 merozoite, day 4; 4 merozoite, day 7; 5 merozoite, day 9; 6 piroplasm. Y-axis: expression level expressed as log_2._
